# The nutritional and health attributes of kiwifruit: a review

**DOI:** 10.1007/s00394-018-1627-z

**Published:** 2018-02-22

**Authors:** David P. Richardson, Juliet Ansell, Lynley N. Drummond

**Affiliations:** 1 DPR Nutrition Ltd., 34 Grimwade Avenue, Croydon, Surrey CR0 5DG UK; 2 Zespri International Ltd., 400 Maunganui Road, Mount Maunganui 3116, Tauranga, New Zealand; 3 Drummond Food Science Advisory Ltd., 1137 Drain Road, Killinchy, 7682 New Zealand

**Keywords:** Kiwifruit, Nutritional composition, Vitamin C, Digestive health, Metabolic benefits

## Abstract

**Purpose:**

To describe the nutritional and health attributes of kiwifruit and the benefits relating to improved nutritional status, digestive, immune and metabolic health. The review includes a brief history of green and gold varieties of kiwifruit from an ornamental curiosity from China in the 19th century to a crop of international economic importance in the 21st century; comparative data on their nutritional composition, particularly the high and distinctive amount of vitamin C; and an update on the latest available scientific evidence from well-designed and executed human studies on the multiple beneficial physiological effects.

Of particular interest are the digestive benefits for healthy individuals as well as for those with constipation and other gastrointestinal disorders, including symptoms of irritable bowel syndrome. The mechanisms of action behind the gastrointestinal effects, such as changes in faecal (stool) consistency, decrease in transit time and reduction of abdominal discomfort, relate to the water retention capacity of kiwifruit fibre, favourable changes in the human colonic microbial community and primary metabolites, as well as the naturally present proteolytic enzyme actinidin, which aids protein digestion both in the stomach and the small intestine. The effects of kiwifruit on metabolic markers of cardiovascular disease and diabetes are also investigated, including studies on glucose and insulin balance, bodyweight maintenance and energy homeostasis.

**Conclusions:**

The increased research data and growing consumer awareness of the health benefits of kiwifruit provide logical motivation for their regular consumption as part of a balanced diet. Kiwifruit should be considered as part of a natural and effective dietary strategy to tackle some of the major health and wellness concerns around the world.

## Introduction

Kiwifruit are a nutrient-dense fruit and extensive research over the last decade on the health benefits of kiwifruit has linked their regular consumption to improvements not only in nutritional status, but also benefits to digestive, immune and metabolic health. The health benefits of consuming fruit are well documented [1]. Kiwifruit are exceptionally high in vitamin C and contain an array of other nutrients, notably nutritionally relevant levels of dietary fibre, potassium, vitamin E and folate, as well as various bioactive components, including a wide range of antioxidants, phytonutrients and enzymes, that act to provide functional and metabolic benefits. The contribution of kiwifruit to digestive health is attracting particular attention owing to a growing body of evidence from human intervention studies. There are several plausible mechanisms of action that are likely to act together including the fibre content and type, the presence of actinidin (a natural proteolytic enzyme unique to kiwifruit which breaks down protein and facilitates gastric and ileal digestion [2, 3]), and other phytochemicals which may stimulate motility [4].

The kiwifruit of commercial cultivation are large-fruited selections of predominantly *Actinidia deliciosa* cv Hayward (green kiwifruit) and an increasing range of gold varieties of various *Actinidia* species. The Hayward cultivar is an oval-shaped berry with a dull brown hairy skin, however, one of its most attractive features is the strikingly beautiful appearance of the bright translucent green flesh interspersed with several rows of small black seeds. As an example of a gold fleshed kiwifruit Zespri® Sungold (*Actinidia chinensis spp*.) have a bright yellow flesh surrounded by a smooth, hairless, bronze-coloured skin. The flesh of the green Hayward cultivar is described as a tangy, sweet and sour combination providing a unique flavour combination, whereas the gold cultivar is described as having a sweet and tropical taste [5, 6].

## History

In the twentieth century, kiwifruit came a long way from being a wild species partially exploited by man to being a commercial crop of international economic importance [7]. Kiwifruit are native to the temperate forests of the mountains and hills of southwest China. Missionaries in the nineteenth century made many contributions to the advancement of botany and the distribution of horticultural plants [8]. The first botanical specimens of *A. chinensis* were sent to Europe by the Jesuit priest Père Pierre Noël Le Chéron d’Incarville around the 1750s and later by Robert Fortune, a plant collector. Robert Fortune was sent to China by the Horticultural Society of London (1843–1845) to “collect seeds and plants of an ornamental or useful kind”, and one of Fortune’s specimens of *A. chinensis* was held at the Royal Botanic Gardens at Kew, London. The first fruits of *A. chinensis* to be seen in Europe were sent, preserved in spirit, to Kew in 1886. Today New Zealand is a major producer of kiwifruit, and all early commercial varieties of kiwifruit plants in New Zealand and around the world can be traced back to a Church of Scotland mission station in Yichang, China, in 1878. Early in the twentieth century, the seeds and plants were regarded as ornamental curiosities with no mention of the edible fruit. The introduction of kiwifruit to New Zealand can be traced to a school teacher, Isabel Fraser, who in 1904 returned from a visit to China with seeds [7]. Around 1922, Hayward Wright, a nurseryman living near Auckland, New Zealand, offered plants in his catalogue, listing the plant as “a wonderful fruiting climber” and promoting it as a highly valuable new fruit because it ripens in the winter over a long period, thus making the fruit a valuable addition to the short supply of winter fruits.

The Hayward cultivar has been sold widely from the late 1930s and the dominance of this cultivar worldwide is now complete. The first commercial orchards and large-scale plantings occurred around this time. Orcharding kiwifruit required brave and courageous decisions as the work was hard, there were no proven patterns of management by growers and agronomic problems were faced as they occurred. World War II and then agricultural and marketing incentives from the 1950s to the present day resulted in the rapid geographical expansion of orchards in New Zealand, Australia, Chile, USA and Europe, mainly Italy, France and Greece. In Italy, the high content of vitamin C gave kiwifruit the reputation of being the “frutto della salute”—the health fruit [8].

The last 100 years have seen the domestication of the kiwifruit from being a wild plant (the so-called “Chinese gooseberry”) to a stage where it is now an important crop in several countries. The name “kiwifruit” was proposed by Turners and Growers Ltd, an exporting firm in Auckland, after the flightless bird, which is endemic to, and often taken as, the emblem of New Zealand. Servicemen were also commonly known as “Kiwis”, and by 1969 the name kiwifruit was well established and accepted.

The process of domestication of kiwifruit is a fascinating and complex story. It includes botanical identification, the collection of seeds and propagating material, cultivation techniques to grow and manage the plant, the management of a dioecious perennial climber, selection of the best cultivars, the commercial discoveries of the cultural conditions affecting yield, harvesting, storage, packing to extend the season and transporting across the globe [8].

Of all the different species of *Actinidia*, the main cultivar of economic importance is *A. deliciosa*, and all the commercial plantings in New Zealand can be traced back to the seeds introduced by Isabel Fraser. The geographic range, the diversity of the wild population and subsequent development of cultivars, including gold and red-fleshed varieties, indicate that the gene pool, mostly sourced from wild types in China, offers many opportunities for breeding programmes for many desirable attributes, including very high levels of vitamin C [5, 9]. Whilst the kiwifruit season requires winter growing, the fruit can be stored very well once harvested and also is produced in both the northern and southern hemispheres. This means that kiwifruit is available throughout the year which is important for those interested in regular consumption for its health benefits [10].

## The nutritional attributes of kiwifruit

Comprehensive and independent data on the nutritional composition of kiwifruit can be found in the USDA National Nutrient Database for Standard Reference [11] and the New Zealand Food Composition Database (NZFCD) [12]. Chemical analyses are conducted on fruit ripened to the “ready-to-eat” state to ensure that the data are reflective of what would normally be consumed. Typically, kiwifruit (*A. deliciosa* and *A. chinensis—*“green” and “gold” cultivars, respectively) are eaten with the skin removed, and hence the analyses shown in Table 1 are completed on the edible flesh portion only. A recent update to this information in the NZFCD now includes nutritional composition of the skin, as there is anecdotal evidence of growing number of consumers who choose to eat the skin, particularly of the gold varieties since it is smoother, thinner, and hairless. Consumption of whole SunGold kiwifruit (including the skin) increases the fibre, vitamin E and folate contents by 50, 32 and 34%, respectively [13].

**Table 1:** Nutritional composition of kiwifruit (Source: USDA National Nutrient Database for Standard Reference Release 28, green and gold raw kiwifruit per 100g [11])

Nutrient	Units/100 g	Green Kiwifruit	Gold Kiwifruit
Proximates			
Water	g	83.1	82.4
Energy	kcal	61	63
Energy	kJ	255	262
Protein	g	1.14	1.02
Total lipid (fat)	g	0.52	0.28
Ash	g	0.61	0.47
Carbohydrate, by difference	g	14.7	15.8
Fiber, total dietary	g	3	1.4
Sugars, total	g	9.0	12.3
Minerals			
Calcium, Ca	mg	34	17.0
Iron, Fe	mg	0.31	0.21
Magnesium, Mg	mg	17	12.0
Phosphorus, P	mg	34	25
Potassium, K	mg	312	315
Sodium, Na	mg	3	3
Zinc, Zn	mg	0.14	0.08
Copper, Cu	mg	0.13	0.103
Manganese, Mn	mg	0.098	0.05
Selenium, Se	µg	0.2	0.44
Vitamins			
Vitamin C, total ascorbic acid	mg	92.7	161.3
Vitamin B1-Thiamin	mg	0.027	< 0.01
Vitamin B2-Riboflavin	mg	0.025	0.074
Vitamin B3-Niacin	mg	0.341	0.231
Vitamin B5-Pantothenic acid	mg	0.183	0.12
Vitamin B6-Pyridoxine	mg	0.063	0.079
Vitamin B9-Folate	µg, DFE	25	31.0
Choline	mg	7.8	1.9
Vitamin B-12	µg	0	0.08
Vitamin A, RAE	µg _RAE	4	1
Vitamin A	IU	87	23
Vitamin E (α-tocopherol)	mg	1.46	1.51
Vitamin K	µg	40.3	6.1
Others			
Carotene, beta	µg	52	14
Lutein + zeaxanthin	µg	122	24
Scientific Name:		*Actinidia deliciosa*	*Actinidia chinensis*
Cultivar		*Hayward*	*SunGold*
USDA NDB No		09148	09520

### Vitamin C

**Fig. 1:**
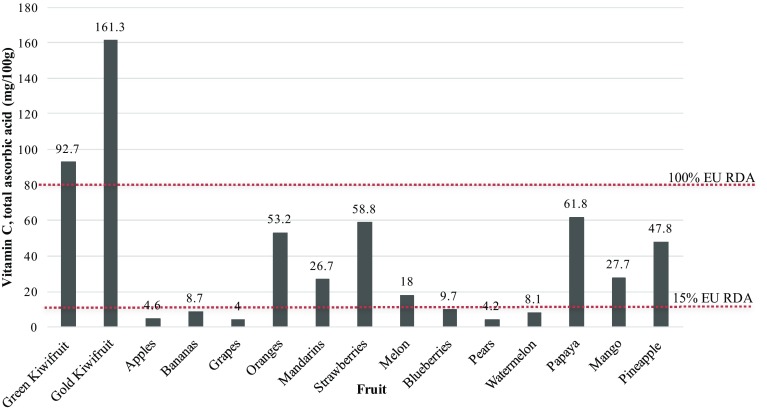
Graph comparing the vitamin C content of kiwifruit with other commonly consumed fruit

The total ascorbic acid content is the most distinctive nutritional attribute of kiwifruit [12]. The levels in the Hayward green cultivar are typically between 80 and 120 mg per 100 g fresh weight [14]. This natural variation of amounts of vitamin C in fruit, including kiwifruit, is due to numerous factors including growing region and conditions, use of fertilisers, maturity at harvest, time of harvest, storage and ripening conditions [15]. In terms of nutritional value, using scoring models that rank and compare the amount of important nutrients present in foods, kiwifruit score well against other fruit. This provides a useful means for communicating those nutritional benefits to consumers [16–18], and should be noted that the high nutrient density score is largely driven by their high vitamin C content [12]. Figure 1 compares the vitamin C content of various fruits to that of Hayward and SunGold kiwifruit cultivars. The SunGold kiwifruit contains 161.3 mg vitamin C per 100 g—almost three times the amount found in oranges and strawberries on an edible flesh weight basis.

From the technical and sensory perspectives, the high ascorbic acid and low tannin content in kiwifruit are thought to explain why the cut fruit does not develop the typical browning reaction that occurs in most other fruits [14].

Vitamin C (ascorbic acid) is an essential dietary nutrient for humans, as we lack the terminal enzyme l-gulono-γ-lactone oxidase in the ascorbate biosynthetic pathway [19]. There is an absolute requirement for Vitamin C for a range of biological functions. Vitamin C is a cofactor of metallo-enzymes necessary for the biosynthesis of collagen, l-carnitine, catecholamine, neurotransmitters, and peptide hormones such as oxytocin [20, 21]. Vitamin C in involved a in the regulation of transcription factors [22]. The strong antioxidant properties of Vitamin C are well documented, it scavenges free radicals and other reactive oxygen and nitrogen species, with a capacity to regenerate other small molecule antioxidants from their respective radicals [23, 24]. Thus, it protects biomolecules such as lipids and DNA against oxidative damage [25, 26].

There is evidence from in vitro, as well as animal and human intervention studies that supports the role of vitamin C in the functioning of the immune system. Leukocytes, which are cells responsible for defending the body against invading pathogens, contain high levels of vitamin C, indicating a vital function in the immune system [27]. These cells include neutrophils, the first cellular responders to inflammatory challenge. Their primary function is to destroy invading microorganisms and thereby prevent systemic infection [28, 29].

A recent Cochrane systematic review [30] upholds the role of vitamin C in improving immune function and reducing the duration of common cold symptoms in the ordinary population. A Gold kiwifruit intervention study showed enhanced plasma vitamin C concentration and reduced severity and duration of upper respiratory infection symptoms in 32 elderly people supplemented with four kiwifruit per day for 4 weeks [31].

An effectively functioning immune system is crucial for maintaining physiological integrity, and the European Food Safety Authority (EFSA) Panel on Dietetic Products, Nutrition and Allergies (NDA) [32] considers that maintaining normal immune function is a beneficial physiological effect. Given the multiple roles of the immune system providing defences against infections and allergic manifestations such as asthma, urticaria and eczema, the specific effect on immune function is required for scientific substantiation of health claims on a food/constituent. The requirements for substantiation of health claims on maintaining normal immune function in a population group considered to be at risk of immunosuppression (e.g., older adults, individuals experiencing stress or engaging in heavy physical exercise, or after exposure to ultraviolet radiation) are provided in the scientific opinion of EFSA [32].

The vitamin C content of green and gold kiwifruit is 92.7 and 161.3 mg per 100 g, respectively [11]. In the European Union, the Reference Intake (RI) for vitamin C for labelling purposes is 80 mg [33]. For “source” and “high” nutrient content claims for vitamin C, the amounts required for the claims are 15% RI, or 12 mg, and 30% RI, or 24 mg, per 100 g, respectively. Hence the levels of vitamin C in kiwifruit qualify them as being high in the vitamin, and eligible for authorised health claims in the European Union (EU) for vitamin C nutrient functions (Table 2). Amongst a background of a large number of antioxidant species, the vitamin C content of kiwifruit has the highest correlation with total antioxidant activity of kiwifruit [34].

**Table 2:** Summary of well-established functions of selected vitamins and minerals under Article 13.1 of the Nutrition and Health Claims Regulation (European Commission 2006) and the proposed wording as shown on the EU Register

Nutrients	Health claims
Vitamin C	Vitamin C contributes to normal collagen formation for the normal function of blood vessels Vitamin C contributes to normal collagen formation for the normal function of bones Vitamin C contributes to normal collagen formation for the normal function of cartilage Vitamin C contributes to normal collagen formation for the normal function of gums Vitamin C contributes to normal collagen formation for the normal function of skin Vitamin C contributes to normal collagen formation for the normal function of teeth Vitamin C contributes to normal energy-yielding metabolism Vitamin C contributes to normal functioning of the nervous system Vitamin C contributes to normal psychological function Vitamin C contributes to normal function of the immune system Vitamin C contributes to the protection of cells from oxidative stress Vitamin C contributes to the reduction of tiredness and fatigue Vitamin C contributes to the regeneration of the reduced form of vitamin E Vitamin C increases iron absorption Vitamin C contributes to maintain the normal function of the immune system during and after intense physical exercise (> 200 mg/day)
Vitamin E	Vitamin E contributes to the protection of cells from oxidative stress
Folate	Folate contributes to maternal tissue growth during pregnancy Folate contributes to normal amino acid synthesis Folate contributes to normal blood formation Folate contributes to normal homocysteine metabolism Folate contributes to normal psychological function Folate contributes to the normal function of the immune system Folate contributes to the reduction of tiredness and fatigue Folate has a role in the process of cell division
Potassium	Potassium contributes to normal functioning of the nervous system Potassium contributes to normal muscle function Potassium contributes to the maintenance of normal blood pressure

High levels of vitamin C in kiwifruit can improve iron bioavailability [35]. Poor iron status remains one of the most common micronutrient concerns globally [36], and is associated with a number of adverse health consequences [37]. In a study of individuals with low iron status [serum ferritin (SF) ≤ 25 µg/L and haemoglobin (Hb) ≥ 115 g/L], eating kiwifruit with an iron-fortified breakfast cereal was found to improve iron status [35, 38]. In this study, 89 healthy women were randomised to receive iron-fortified breakfast cereal, milk and either two Zespri gold kiwifruit or one banana at breakfast every day for 16 weeks. After 16 weeks, median serum ferritin significantly increased from 17.0 µg/L at baseline to 25.0 µg/L, compared to the banana group, which had a median serum ferritin level of 16.5 µg/L at baseline that rose to 17.5 µg/L at the end of the study (*P* < 0.001). Importantly, the 10 µg/L increase in serum ferritin in the women who ate kiwifruit increased levels to within the normal reference range of 20–160 mg/L. Additionally, median soluble transferrin receptor concentrations significantly decreased by − 0.5 mg/L for kiwifruit versus 0.0 mg/L for banana (*P* = 0.001) [35, 38].

Significant proportions of the population around the world, including the UK [39], have very poor fruit and vegetable intakes that result in suboptimal vitamin C status. The maintenance of the body pools and of plasma and cellular vitamin C concentrations are considered criteria for establishing the requirements for vitamin C based on the assumption that saturation of body pools and plasma concentrations are associated with fulfilling the essential functions of vitamin C in the body [26]. Saturating plasma levels, now considered to be associated with optimal health and wellbeing, are found in only around 20% of the normal, healthy population. Carr et al. [40] showed that consuming kiwifruit had a strong effect on plasma and muscle [23] vitamin C levels. To measure the contribution of gold kiwifruit to dietary vitamin C intake, plasma vitamin C levels were measured in a group of 14 male students with low vitamin C status (average baseline plasma, 38 mM). Participants were asked to consume half a kiwifruit per day for 4 weeks, two kiwifruit per day for 6 weeks and finally three kiwifruit per day for 4 weeks. The addition of as little as half a kiwifruit per day resulted in a significant increase in plasma vitamin C. However, one kiwifruit per day was required to reach what are considered to be healthy levels [40]. At two kiwifruit per day, plasma levels approached saturation, with no further increases with three kiwifruit per day. This observation was confirmed by increased urinary output of vitamin C at two kiwifruit per day, which coincided with plasma levels reaching around 60 mM. These results confirmed the pharmacokinetic data of Levine et al. [41] and indicated that plasma vitamin C levels in humans saturate at an intake of about 200 mg/day. This is equivalent to eating approximately two kiwifruit per day.

Furthermore, vitamin C and increased consumption of fruits and vegetables have been shown to be associated with enhanced feelings of wellbeing and vitality [42–45]. It is well established that fatigue and lethargy are common early symptoms of subclinical vitamin C deficiency and can be resolved with vitamin C supplementation [46]. The effects of vitamin C on fatigue are likely explained by its in vivo function as an enzyme cofactor for the synthesis of important biomolecules such a dopamine, neurotransmitters and hormones synthesised by the nervous system and adrenal glands [47].

### Vitamin E

Kiwifruit contain relatively high levels of vitamin E [12, 48], compared to other commonly consumed fruit. SunGold and green kiwifruit contain 1.40 and 1.46 mg per 100 g [11], respectively, of the main form, α-tocopherol present in the flesh [49]. These levels are sufficient to permit the use of nutrient function claims for Vitamins E in the EU (Table 2). Fiorentino et al. [49] showed that α-tocopherol is found in the flesh of kiwifruit, possibly associated with cell membranes and therefore potentially bioavailable. Fiorentino et al. [49], also identified a new form of vitamin E in kiwifruit, δ-tocomonoenol, noting that its radical scavenging and antioxidant capacity contributed to the total antioxidant activity of kiwifruit. Studies showing that the consumption of both green and gold kiwifruit correlates with increased plasma vitamin E concentrations, suggest the vitamin E in kiwifruit is bioavailable [31, 50].

### Folate

**Fig. 2:**
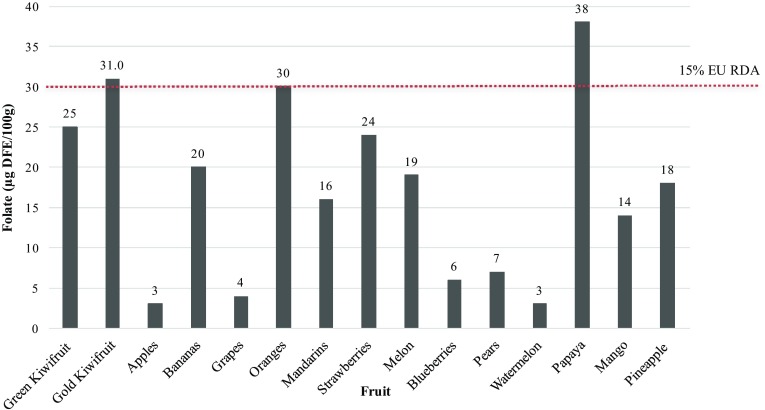
Graph comparing the folate content of kiwifruit with other commonly consumed fruit

Kiwifruit are often referred to as being a good source of dietary folate. The folate content of kiwifruit green and gold cultivars compared with other fruits are shown in Fig. 2. The folate content of 31 µg per 100 g in gold kiwifruit meets the criteria of EU Regulation to make a “source” claim as it exceeds the 15% of the Reference Intake of 200 µg/day. In other countries, where the recommended daily intake is often higher (e.g., 400–500 µg/day in Nordic counties, 400–600 µg/day in the USA Australia and NZ), such nutrient content claims cannot be made. The authorised health claims in the EU for folate nutrient functions are shown in Table 2.

As folate is extremely labile and its presence in green leafy vegetables is easily destroyed by cooking, fresh kiwifruit can make a useful contribution to the total diet, especially during pregnancy when it is difficult to meet folate requirements. During pregnancy, folate requirements are 600 µg/day, which can be safely achieved through the use of conventional foods, foods with added nutrients and food supplements [51].

### Potassium

**Fig. 3:**
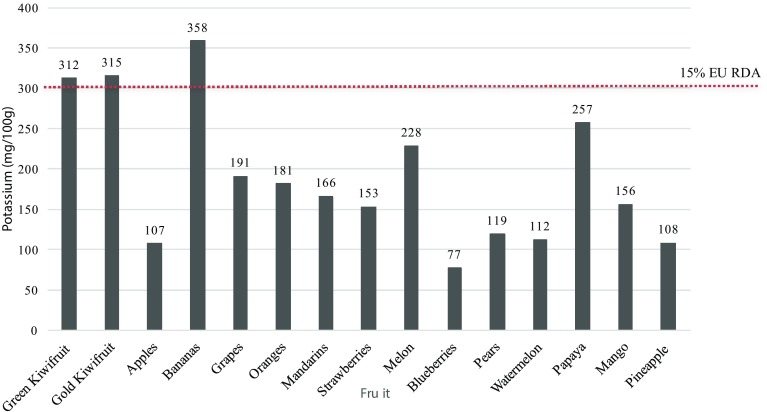
Graph comparing the potassium content of kiwifruit with other commonly consumed fruit

Green and gold kiwifruit are good sources of potassium, containing typically around 301–315 mg per 100 g. These amounts are sufficient to meet the criteria of EU Regulation (EC) no. 1924/2006 on nutrition and health claims made on foods to make a natural “source” claim, as it exceeds the 15% of the Reference Intake of 2000 mg/day. The authorised nutrient function health claims in the EU for potassium are shown in Table 2. The potassium content of kiwifruit compared to other fruit is shown in Fig. 3. In other countries, where the recommended daily intake is often higher, such content claims cannot be made.

Fresh foods such as fruits and green vegetables are generally good sources of potassium and low in sodium. The sodium content of kiwifruit is only 3 mg per 100 g and can be described as naturally low in sodium. The sodium to potassium (Na^+^/K^+^) ratio of kiwifruit is consistent with recommendations to increase potassium intake through increased consumption of fruit and vegetables, and is amongst the more favourable Na^+^/K^+^ balance of selected fruits [52]. Studies have provided evidence that potassium rich diets or interventions with potassium can lower blood pressure, especially in individuals with hypertension [53, 54], however, more recently the dietary Na^+^/K^+^ ratio has been shown to be more strongly associated with an increased risk of hypertension and CVD-related mortality than the risk associated with either sodium or potassium alone [55, 56].

### Dietary fibre

The dietary fibre of kiwifruit comes almost entirely from the plant cell walls, and particularly the polysaccharides that form the major structural components of these walls. Kiwifruit contain about 2–3% of fresh weight non-starch polysaccharides [48] that make up the fruit cell walls, providing a valuable contribution of both soluble and insoluble fibre to the diet. Analysis of dietary fibre of green and gold kiwifruit has shown they comprise about one-third soluble and two-thirds insoluble fibres, although kiwi gold fruit contain considerably less total fibre than green [57]. The soluble fibre fraction contains almost exclusively pectic polysaccharides, whereas the insoluble fibre is mostly cellulose and hemicelluloses.

Changes occur in the composition and structure of kiwifruit cell walls during development and ripening. These structural changes in cell wall polysaccharides are reviewed in detail by Sims, Monro [58]. Cell wall polysaccharides are generally resistant to digestion and absorption in the human small intestine and are considered to be delivered to the colon in a chemically unaltered state. However, even minor chemical or structural changes can impact on the physicochemical properties and fermentability that determine their impact on health.

In the hind-gut, the physiological benefits of fibre are believed to arise from the products of bacterial fermentation of the soluble fibre, and from the physicochemical properties of any fibre that remains unfermented [59, 60]. Among the most important physicochemical properties of kiwifruit fibres are the hydration properties, which include water retention, capacity and swelling, viscosity (which requires solubility), and properties that depend on the size, shape and porosity of undigested particles. Water retention is physiologically relevant because it influences transit time, faecal bulk, stool consistency and other functional benefits [60]. The high swelling and water retention of kiwifruit fibre in comparison with other forms of dietary fibre such as wheat bran, commercial preparations of sugar beet fibre and apple fibre, accentuate the value of consuming kiwifruit as a natural whole product that has had minimal processing. Kiwifruit dietary fibres are susceptible to fermentation, and so many provide benefits through the production of the short chain fatty acids [58]. Future studies on the mechanisms by which kiwifruit dietary fibres, as part of a balanced diet, modulate digestion processes and act as a substrate for beneficial colonic microbiota, may aid understanding of the actions of fibre in the gut [61] and its beneficial effects on human health.

### Sugars

As kiwifruit develop and ripen, the concentrations of chemical components in the tissue change. The most marked change in the physiology of the fruit during ripening leads to a rapid decrease in starch concentration and a consequent increase in fructose and glucose. Kiwifruit tissue is very hard while the fruit is developing on the vine, but flesh firmness decreases during the later stages of development [14]. Fortunately, kiwifruit that are physiologically mature but have barely started to ripen can be harvested and will continue to ripen successfully off the vine. Cool storage immediately after harvest reduces the rate of ripening. It is these particular characteristics of kiwifruit that allow producing countries such as New Zealand to store unripe fruit and ship to it distant markets over an extended period. Suitable indicators of maturity for kiwifruit are used to ensure that fruit reaches an appropriate stage of development before harvest. A “maturity value” is important, and three changes in kiwifruit are taken into account—decreasing flesh firmness, conversion of starch to sugar and soluble solids concentration (to measure sugar concentration) are all used to provide an accurate assessment of final eating quality. The predominant sugars present in *Actinidia* are glucose and fructose with a small amount of sucrose present when the fruit is ripe and ready-to-eat. The amount of total sugars and ratios of these sugars vary not only as a function of maturity but also with the variety of kiwifruit [62, 63]. The ratio of fructose: glucose is important in terms of digestive health and preferably should be around 1:1 to reduce symptoms of gastrointestinal discomfort, such as bloating, caused by rapid fermentation by gut bacteria.

Interestingly, as they ripen, many fruits undergo a marked decrease in chlorophyll content, and carotenoids and anthocyanins become dominant. These visual changes indicate the stage of ripeness. On the other hand, in green kiwifruit there is little if any decrease in chlorophyll content and the internal colour remains an attractive bright green when fruit are “eating ripe”. As kiwifruit begins to ripen, starch concentration decreases from 6% of fresh weight to trace amounts, and total sugars increase to 12–15%. The concentration of soluble solids also increases to reach a plateau of 14–16% before fruit is eating ripe.

Understanding the factors affecting the rate of ripening is of considerable commercial importance for fruit quality. In fruit that is ready for consumption the sugars provide the appealing sweet flavour of kiwifruit, which is balanced by the organic acid composition [62, 63].

From a physiological perspective, the sugar content of kiwifruit, like all fruit, may potentially influence the management of blood sugar levels following their consumption, however current research suggests the glycaemic response effects of kiwifruit as a whole food are potentially different to that which could be expected of individual components [64]. Interestingly the glycaemic index (GI) of kiwifruit is relatively low (green kiwifruit, 39.3 ± 4.8 and gold kiwifruit, 48.5 ± 3.1 [65]). The low GI value of kiwifruit is observed in both healthy human subjects and those with Type 2 diabetes [66]. The importance of managing postprandial blood sugar levels is covered in the section on metabolic health.

### Antioxidants

In addition to the various nutrients in kiwifruit described above, for which there are dietary intake recommendations and well described physiological functions, kiwifruit contain a complex network of minor compounds that may also be associated with beneficial physiological functions. Various *Actinidia* species have been extensively analysed for their antioxidant chemical profiles [67–71]. As well as vitamins C and E, the other antioxidants include the carotenoids lutein, zeaxanthin and β-carotene, chlorophylls, quinic acid, caffeic acid glucosyl derivatives, β-sitosterol, chlorogenic acid, phenolics, including flavones and flavonones, to name but a few [72–75]. The antioxidant capacity of kiwifruit constituents has been measured by means of various in vitro chemical assays that monitor the quenching, scavenging or retarding of free radical generation [6]. For example, the total antioxidant capacity of kiwifruit was reported to be higher than apple, grapefruit and pear, but less than raspberry, strawberry, orange and plum [76, 77]. While these in vitro studies indicate that the various antioxidants are capable of preventing or delaying some types of cell damage from the unstable free radicals created every day during normal metabolism, the detailed mechanism of how this translates to effects in vivo which are directly linked physiological changes is yet to be fully understood [78]. In a number of human studies, beneficial changes to biomarkers of CVD, have been attributed to the antioxidant compounds present in kiwifruit [79–85]. The stability of antioxidants during simulated in vitro gastrointestinal digestion [86, 87], and their bioaccessibility/bioavailability [88] provide supportive evidence for the potential for physiological effects of the antioxidants in kiwifruit. There is significant variation in the types and levels of antioxidant compounds and total antioxidant activity both between *Actinidia* species, and as a function of extraction solvent [73–75]. Several studies have explored the influence of growing practices and region on the activity of bioactive and antioxidant compounds in kiwifruit. Park et al. [89] found generally higher, but not consistently significant, levels of bioactive compounds in organically grown kiwifruit, whilst in an Italian study, the geographical location of orchards did not significantly influence vitamin C or polyphenolic contents [90].

Although there are no dietary intake recommendations for antioxidants in general, the scientific data suggest that eating kiwifruit has the potential to inhibit oxidative and inflammatory processes, although the supporting data for antioxidant activities are more substantial than those related to the kiwifruit’s potential anti-inflammatory activities. The results of human studies of the antioxidant efficacy of kiwifruit are inconsistent owing to differences in experimental protocols, the cultivar of kiwifruit used, the amount and duration of the study as well as the biomarkers used [6]. Kiwifruit could undoubtedly be a useful dietary vehicle for delivering antioxidant nutrients and other phytonutrients. Future studies on kiwifruit will explore the bioavailability, metabolism, tissue distribution and biological effects of kiwifruit constituents on relevant disease markers. The emerging evidence could provide the basis for improved dietary strategies for achieving dietary antioxidant and anti-inflammatory health benefits in humans [91].

### Actinidin and minor proteins

Kiwifruit contain several unique proteins and the cysteine protease actinidin, the most abundant protein in kiwifruit, of interest for their bioactive potential.

The characterisation and biochemical properties of actinidin have been extensively studied [92, 93], and more recently its potential role in human health [94, 95]. Actinidin is active over a wide range of pH, including those of the GI tract [96] thus having the potential to influence protein digestion, and intestinal permeability [97]. In contrast to potential benefits (see Digestive health), actinidin is also the major kiwifruit allergen [90, 98]. Green and gold kiwifruit have been known to cause allergic reactions ranging from mild symptoms localised to the oral mucosa in the majority of individuals to anaphylactic reactions, particularly in children [99]. Very little information is available in the literature on the prevalence of kiwifruit allergy, and human intervention studies with kiwifruit have shown that kiwifruit are well tolerated without any adverse side effects [35, 50, 84, 100]. The magnitude and patterns of reactivity to kiwifruit allergens appears to vary with ethnic/geographical/cultural differences, age of subjects and other clinical characteristics of individuals exposed to kiwifruit [6]. Lucas, Atkinson [101] have provided a detailed review of unresolved issues regarding kiwifruit and have suggested requirements to be met prior to designation of allergens to a database. Processing may diminish the risk of allergic symptoms in those with allergies to raw kiwifruit [102, 103].

Kiwellin is another protein in kiwifruit, that as a function of ripening stage and postharvest treatment of the fruit is susceptible to actinidin activity, producing the peptide kissper, and and KiTH [104, 105]. Kissper is of particular interest for human health as it displays a range of beneficial activities, including anti-inflammatory response, reducing oxidative stress at the GI mucosal interface [106], and pH-dependent and voltage-gated pore-forming activity, together with anion selectivity and channelling [4]. This suggests that kissper is a member of a new class of pore-forming peptides with potential beneficial effects on human health, including a potential effect on gastrointestinal physiology [4].

## Digestive health

Early Chinese pharmacopoeia from the Tang Dynasty onwards (AD 618–907) list a whole variety of medicinal uses for “mihoutao” fruit, the Chinese name generally used for *Actinidia* species, including aiding digestion, reduction of irritability and curing of dyspepsia and vomiting.

Functional gastrointestinal disorders (FGIDs) are common and distressing [107]. FGIDs include functional dyspepsia (FD) and irritable bowel syndrome (IBS), affecting an estimated 3–28% of the global population [108], particularly the elderly and women, and may severely affect the individual’s quality of life and wellbeing [107, 109]. Upper gastrointestinal disorders include gastric reflux, stomach ache, delayed gastric emptying, nausea and vomiting, and lower gastrointestinal disorders include constipation, indigestion, bloating and diarrhoea. Current interventions for FGIDs include lifestyle and dietary modifications as well as pharmacological interventions targeting pain, motility, laxation and the gut microbiota [108].

The worldwide growth in the incidence of FGIDs has created an immediate need to identify safe and effective food-based interventions. For example, constipation may be present in up to 29% of the population, depending on the definitions used [110–112]. Food ingredients such as psyllium and wheat bran are the most studied for maintaining a healthy gut and to manage abdominal discomfort. Additionally, it is generally regarded that adequate intakes of fibre-rich fruits and vegetables daily with sufficient water will prevent constipation. Whole green kiwifruit have been used and promoted for many years to maintain abdominal comfort [113] and have been studied more recently under controlled settings [114, 115]. The components found in kiwifruit have been shown to increase faecal bulking and softness and enable better lubrication, assisting the propulsion of content along the colon [116, 117].

It is thought that the unique combination of soluble and insoluble fibres, polyphenols and actinidin, present in kiwifruit, confers the gastrointestinal benefits, improvements in laxation and reduction of abdominal discomfort, both in individuals with either constipation-predominant irritable bowel syndrome (IBS-C) and in normal healthy people suffering from constipation without reported side effects. The putative mechanism of kiwifruit on maintenance of normal GI function has recently been reviewed [95]. The review discusses the physiological functions of the digestive system, the pathophysiological mechanisms behind functional constipation, a summary of the work covering the effects of green kiwifruit on the gut as well as hypothetical mechanisms behind the gastrointestinal effects of green kiwifruit.

Lack of dietary fibre is a contributing factor in people with constipation [118], and both soluble and insoluble fibres can add bulk, increase water retention in the colon [119, 120] and change faecal consistency [121, 122]. Dietary fibre can also decrease transit time [122, 123]. Soluble dietary fibres are the main substrate for the microflora in the GI tract [60]. When setting the Dietary Reference Value (DRV) of 25 g /day for dietary fibre, the EFSA NDA Panel used the role of fibre in bowel function as the most suitable criterion [124]. Consuming 2 green kiwifruit per day would provide approximately 6 g of fibre (24% DRV), therefore, depending on habitual dietary fibre intake this may be a significant contribution to the total daily intake. Kiwifruit typically contain about two-thirds insoluble fibre, and one-third soluble fibre [125], and as previously mentioned, kiwifruit fibre has an impressive water retention capacity [57, 58]. In the native state, the capacity of kiwifruit fibre to swell, defined as the volume fibre has in water after passively settling [126], is more than six times higher than that of apple fibre, and one and a half times higher than psyllium [58], but is significantly reduced when subjected to processing conditions such as dehydration [127]. Feeding studies in pigs [128, 129] as well as observations in human studies [114, 115, 130] have demonstrated that feeding kiwifruit increases water retention and faecal bulking, however animal studies suggest the pectic substances of kiwifruit are highly susceptible to fermentation in the hind-gut [131, 132]. Such fermentation may produce short-chain fatty acids capable of stimulating colonic motility [133] and contribute to the effects of kiwifruit, however the role of kiwifruit fibre in human digestive function is yet to be fully understood. In contrast, but consistent with earlier findings of changes associated with processed kiwifruit, the fibre of a dried kiwifruit product consumed as a part of a mixed fibre diet, did not demonstrate a significant contribution to faecal bulking in the rat [131]. A reduction in GI transit time has been linked to actinidin [128]. Although a considerable proportion of short chain fatty acids have recently been shown to be derived from the fermentation of non-dietary gut materials [134], kiwifruit fibre may also contribute to favourable changes in the human colonic microbial community [135] and their metabolites [136] which are associated with intestinal health [137].

The proteolytic enzyme actinidin from green kiwifruit has been shown in in vitro studies to aid protein digestion both in the stomach and small intestine [2, 3]. For example, a range of common protein sources derived from soy, meat, milk and cereals were incubated with a kiwifruit extract containing actinidin and pepsin at pH 1.9 (a simulation of gastric digestion in humans) [3]. Results in this gastric digestion model showed that for milk, soy and meat protein sources, the presence of kiwifruit extract enhanced digestion to a greater extent than pepsin alone [13]. Likewise, in an in vitro, small intestine digestion model, actinidin-containing kiwifruit extract was particularly effective in improving the digestion of whey protein, zein, gluten and gliadin [2]. These studies suggest that actinidin may assist with protein digestion in the gastric and ileal regions, that may be of benefit particularly to individuals with compromised digestive function [138]. Under in vitro conditions, gastric lipase remained active, however actinidin effectively inactivated amylase suggesting that when cooked starch is consumed together with kiwifruit it is possible that starch digestion may be retarded [139].

There is growing evidence that kiwifruit have beneficial effects on digestive health and general wellbeing, a potentially important characteristic in the light of the increasing proportion of the elderly population in ageing societies that experience impaired bowel function, changes in gastrointestinal function [138], and gastrointestinal discomfort.

Table 3 summarises the findings from human clinical trials with fresh green kiwifruit. The daily consumption of two kiwifruit was found to increase stool frequency, including the number of complete spontaneous bowel motions per week, reduce gastrointestinal transit time and improve measures of intestinal comfort. These early human studies [50, 114, 130, 140–142] were carried out in different countries and included different study populations (e.g., differing in age, health status), and the lack of a common protocol may have led to results that were not applicable to the larger normal healthy population. Most studies consider the effects of prolonged kiwifruit consumption, however recently Wallace et al. [143] investigated the acute effects of green kiwifruit on gastric emptying following consumption of a steak meal, using a computerised SmartPill™, and measures of digestive comfort. Although the SmartPill™, did not provide reliable data following the meal event, there was a significant reduction in bloating and other measures of gastric discomfort [143]. A multi-country, randomised, cross-over, controlled human intervention study is currently underway to evaluate further the effects of green kiwifruit on digestive function [144]. Changes in bowel function in the general population such as reduced transit time, more frequent bowel movements, increase faecal bulk or softer stools are considered by EFSA to be beneficial physiological effects, provided they do not result in diarrhoea [32]. Similarly, reducing gastrointestinal discomfort [e.g., bloating, abdominal pain/cramps, borborygmi (rumbling)] are considered appropriate outcome measures in human studies that include the use of validated questionnaires on severity and frequency of symptoms. The EFSA Panel on Dietetic Products Nutrition and Allergies (NDA) [32] has also stated that IBS patients or subgroups of IBS are generally considered an appropriate study group to substantiate health benefits on bowel function and GI discomfort.

**Table 3:**
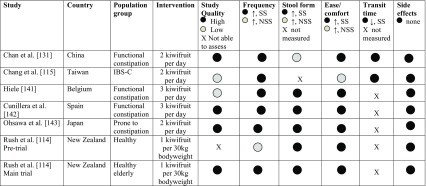
Summary of findings from human studies with fresh green kiwifruit for digestive health

Fermentable Oligosaccharides, Disaccharides, Monosaccharides and Polyols (FODMAPs) are rapidly fermentable, poorly absorbed carbohydrates found in food that can cause digestive discomfort, especially for people with IBS [145]. The action of FODMAPS is likely via multiple pathways [146], and includes the release of gases from the bacterial fermentation of oligosaccharides and the proportion (if any) of malabsorbed fructose, polyols, and lactose [147]. Symptoms associated with FODMAPs include abdominal bloating, pain, cramps, excessive flatulence and altered bowel habit [146]. Low FODMAP diets are effective in the treatment of functional gastrointestinal symptoms [148, 149].

Kiwifruit are certified as low FODMAP fruits by the Monash University low-FODMAP diet digital application (https://www.monashfodmap.com/i-have-ibs/get-the-app/), based on their relatively low proportions of fructose and fructans per single fruit serve. A recent pilot study demonstrated that the consumption of two green kiwifruit is not associated with clinically significant evidence of colonic fermentation as shown by hydrogen and methane on breath testing [150], lending support for the low FODMAP status for kiwifruit.

## Metabolic health

Metabolic abnormalities such as dyslipidaemia [increased blood total cholesterol (TC), low density lipoprotein cholesterol (LDL-C), triglycerides (TG), lower high density lipoprotein cholesterol (HDL-C)], hypertension, vascular inflammation, abnormal glucose metabolism and haemostatic disorders all play important roles in the pathophysiology of the major causes of morbidity and mortality such as diabetes, cardiovascular disease (CVD), stroke and dementia [151–153]. A number of studies have investigated the effects of green and gold kiwifruit on some of these metabolic markers, including the effects of kiwifruit on glucose and insulin balance, and on bodyweight maintenance and energy homeostasis.

Green and gold kiwifruit have clinically measured glycaemic indices (GIs) of 39 and 48, respectively [65], which puts them in the GI “low” category (GI < 55). The glycaemic response to a fruit depends not only on GI, but also the amount of carbohydrate consumed in the fruit. As kiwifruit contains only about 12% available carbohydrate and a low GI; the impact kiwifruit produces on plasma glucose levels is low enough for the fruit to be suitable in managing diets for people with reduced tolerance to glucose. The fibre content of kiwifruit may cause a delay in carbohydrate digestion and absorption by way of swelling action that reduces the rate of glucose diffusion [57, 127]. This reduction in glycaemic response by 200 g kiwifruit (approximately two fruits) has been demonstrated in a human intervention study conducted by Mishra et al. [154]. The authors concluded that the low in vivo glycaemic impact could partly be attributed to the carbohydrate in kiwifruit being fruit sugars (fructose) and partly to the non-digested fibre components reducing the rate of intestinal processes such as digestion, sugar diffusion and mixing of intestinal contents. This partial substitution of starch-based staple foods, such as a high carbohydrate breakfast cereal, with kiwifruit could be an effective and healthy way to improve glucose homeostasis [154]. Further exploration of this effect was investigated by Mishra et al. [64] to better understand the role of non-sugar components in kiwifruit in modulating glycaemic response. Kiwifruit consistently reduced the amplitude of the glycaemic response of participants following a series of wheat-based cereal meals adjusted to match the available carbohydrates from kiwifruit leading the investigators to conclude that components other than the available carbohydrate in kiwifruit, such as cell wall remnants or phenolic compounds, may be involved in the improved glycaemic response to co-ingested foods [64]. The energy value of foods is also an important dietary aspect in managing risk factors for metabolic syndrome. Using an in vivo–in vitro model that determines the available energy (AE) content based on ATP yield at the cellular level [155], Henare et al. [156] found the AE of green and gold kiwifruit was significantly less than that predicted by the traditional Atwater system, suggesting kiwifruit are useful in dietary weight management strategies. Further studies will explore the use of kiwifruit as an effective dietary strategy to reduce postprandial hyperglycaemia while at the same time increasing the amount of essential nutrients consumed.

Regular consumption of green and gold kiwifruit can also affect beneficially certain physiological biomarkers, particularly in individuals with metabolic abnormalities related to major causes of morbidity and mortality, such as diabetes, cardiovascular disease (CVD), stroke and dementia [157]. For example, Chang, Liu [158] investigated the effects of two kiwifruits on the lipid profile, antioxidants and markers of lipid peroxidation in hyperlipidaemic adult men and women in Taiwan. After 8 weeks of the intervention, the HDL-C concentration was significantly increased, whilst the LDL-C/HDL-C ratio and TC/HDL-C ratio were significantly decreased. Vitamin C and vitamin E, the antioxidant nutrients, together with plasma antioxidant status, also increased significantly in fasting blood samples.

Gammon et al. [100] found that consumption of two green kiwifruit per day for 4 weeks favourably affects plasma lipids in a randomised controlled trial in 85 normotensive and pre-hypertensive hypercholesterolaemic men compared with the consumption of a healthy diet alone. Small, but significant, differences occurred, including an increase in HDL-C and a decrease in TC: HDL-C ratio and TG’s. There were no significant differences, however, between the two interventions for plasma TC, LDL-C, insulin, high-sensitivity C-reactive protein (hs-CRP), glucose and blood pressure (BP). In a further exploration of the study, no beneficial effects on markers of cardiovascular function, or on BP were noted [159].

In 2012, Karlsen et al. [80] demonstrated that intake of three kiwifruit per day for 3 weeks promoted pronounced anti-hypertensive effects, as well as antithrombotic effects in male, middle-aged and elderly smokers. The authors commented that this dietary approach may be helpful in postponing pharmacological treatment in individuals with high-normal blood pressure or hypertension. From a further randomised, controlled study over a period of 8 weeks, Svendsen et al. [79] concluded that among men and women aged between 35 and 69 years with moderately elevated BP, the intake of 3 kiwifruit added to the usual diet was associated with lower systolic and diastolic 24-h BP compared with one apple a day. The authors observed these results were in contrast those of Gammon et al. [159], noting the differences in study population criteria (normotensive [159] versus moderately elevated BP [79]) may have been a contributory factor. Although Svendsen et al. [79] found no differences in measures of endothelial function in their study, they suggested that an increase in plasma antioxidant status (lutein), and in increased dietary potassium, resulting from the kiwifruit intervention, could be an explanation for the improvements in BP observed.

In vitro studies on antioxidant and fibrinolytic activities have also indicated the potential cardiovascular protective properties of kiwifruit extracts [160]. Evidence that consumption of kiwifruit can modulate platelet reactivity towards collagen and ADP in human volunteers was provided in a study by Duttaroy, Jørgensen [84]. The authors concluded that kiwifruit may have the potential to increase the effectiveness of thrombosis prophylaxis.

Habitual intakes of high levels of fruits and vegetables have long been associated with beneficial effects that lower the risk of chronic diseases, including CVD in humans [161]. The presence of antioxidant components such as vitamin C, vitamin E, polyphenols [162], a favourable Na^+^/K^+^ ratio [52], and other bioactive components in kiwifruit could explain their beneficial physiological effects [157].

## Concluding remarks

This review highlights the nutritional attributes and health benefits of green and gold kiwifruit. The nutritional composition, particularly the high amount of vitamin C, supports its position as a highly nutritious, low energy fruit. With the plethora of man-made, processed health foods available to the consumer, one aspect that sets kiwifruit apart is that it is a natural, whole food. Nature compartmentalises many bioactive and nutritional components within the complex structure of cell walls, cells and the matrix in between. Human digestion interacts with fresh whole foods to break down the structures and digests the complex carbohydrates slowly. Many health care professionals now recognise whole foods are ideal for the release and delivery of nutrients and health components to various locations along our digestive tract.

There is a growing body of evidence to support the beneficial effects of kiwifruit in gastrointestinal function in healthy individuals as well as in individuals with constipation and other gastrointestinal disorders [143, 144, 163], and recognition for the role of kiwifruit in their management [164]. This presents an evidence-based opportunity for health care professionals to adopt dietary recommendations, and for consumers to recognise the impact of diet, in particular whole foods, on specific body function, and their health and well-being. Green and gold kiwifruit are well characterised and the mechanisms of action for the benefits on gastrointestinal function and modulation of glycaemic responses now being better defined.

Overall, the scientific evidence for the health benefits of kiwifruit needs to be expanded through the conduct of well-designed and executed human intervention studies that clearly define the study populations, the amount and duration of kiwifruit consumption and the specific beneficial physiological effects. A greater understanding of the mechanisms of action of kiwifruit and its bioactive constituents in promoting health also needs to be fully elucidated.

The increased research data identifying the nutritional and health benefits of kiwifruit and their growing consumer acceptance as a part of a balanced diet, will undoubtedly offer opportunities to tackle some of the major health and wellness concerns around the world.
